# Reproductive Ecology of *Prochilodus brevis* an Endemic Fish from the Semiarid Region of Brazil

**DOI:** 10.1100/2012/810532

**Published:** 2012-05-03

**Authors:** Liliane de Lima Gurgel, José Roberto Verani, Sathyabama Chellappa

**Affiliations:** ^1^Programa de Pós-Graduação em Ecologia e Recursos Naturais, Universidade Federal de São Carlos, Rodovia Washington Luiz, km 235, 13565-905 São Carlos, SP, Brazil; ^2^Departamento de Oceanografia e Limnologia, Universidade Federal do Rio Grande do Norte (UFRN), Praia de Mãe Luiza, s/n, 59014-100 Natal, RN, Brazil

## Abstract

The commercially important migratory fish *Prochilodus brevis* is from the Neotropical region, and understanding the reproductive ecology of this potamodromous fish is essential for its conservation and management. This study investigated the length-mass relationship, sex ratio, length at first gonadal maturity, gonadal development stages, gonadosomatic index, condition factor, and reproductive period of *P. brevis*. Temporal distribution of rainfall, temperature, dissolved oxygen concentration, pH, and electrical conductivity of the water were related to the reproductive period of this fish. Rainfall seems to be the main environmental factor which modulates changes in limnological parameters and the timing of the spawning period of this fish. *P. brevis* migrates into lower reaches of the river to feed during the dry season and returns to the upper reaches during the rainy season to spawn. Inadequate facilities for migration create obstacles for spawning success of this ecologically important fish.

## 1. Introduction

Brazil has more endemic freshwater fish species than any other country in the world, besides several distinct biomes rich in animals and plants, such as the Caatinga. This biome covers an area of 734,478 Km² with more than twenty million inhabitants and is considered as one of the most densely populated semiarid regions in the world. Such proportions, in addition to innumerable unique characteristics, call for systematic scientific exploration of its fauna, flora, water and mineral resources as well as ecological and climatic aspects. The semiarid region of Brazil is one of six large climatic zones, showing peculiar characteristics marked by wide variations in rainfall [1]. 

The climatic conditions associated to current global environmental changes and the impermeable nature of the crystalline subsoil are important factors that characterize the drainage network of semiarid northeastern Brazil [2]. As a result of evolutionary processes, the freshwater species that occur in this region reveal the presence of highly specialized fauna, where only the fittest have survived [3]. 

Fish belonging to the order Characiformes are currently distributed throughout the Neotropical region, with a high proportion of detritivorous fish species from the two families Prochilodontidae and Curimatidae. These families include stocks of important fish species which account for approximately fifty percent of the community biomass of some regions [4, 5]. The migratory endemic fish species of the genus *Prochilodus *are distributed in the main South American drainage basins [6]. 

Species of the genus *Prochilodus* are considered as important ecological components of the South American rivers. The effects of removing a dominant migratory detritivorous species, such as *Prochilodus mariae*, on the functioning of the Las Marias ecosystem in the Orinoco Basin were investigated [7]. The absence of this species caused changes in the metabolism and organic carbon flow of this ecosystem, leading to total degradation of the river.

*Prochilodus brevis* is an endemic fish species of the Brazilian semiarid region [8, 9] and is regionally known as curimatã. Although it has considerable economic importance in northeastern Brazil, its reproductive ecology is scarcely known. In the juvenile phase, *P. brevis* feeds on plankton in the lower reaches of the river and gradually becomes iliophagous in the adult phase. It is a rheophilic fish which migrates during the rainy season for several kilometers to the headwaters of the river to spawn [10]. The construction of reservoirs and predatory fishing of *P. brevis*, mainly during the spawning season when females and males are mature, puts their survival at risk, which could affect the functioning of the ecosystems in the future. Furthermore, global climatic changes have been modifying the rainfall regime of the semiarid regions, altering the reproductive process of the fish species.

Considering the importance of *P. brevis* for the semiarid region of northeastern Brazil, the aim of the present study was to characterize its reproductive strategy. The length-weight relationship, sexual proportion, size at first gonadal maturity, gonadal maturation stages, gonadosomatic index (GSI), and condition factor (*K*) were investigated. The reproductive period of *P. brevis* was correlated to environmental variables, such as rainfall, water temperature, electrical conductivity, pH, and concentration of dissolved oxygen.

## 2. Materials and Methods

### 2.1. Study Area

The freshwater ecosystems in the state of Rio Grande do Norte, Brazil, are composed of seven drainage basins. The largest is the Piranhas-Assu hydrographic basin, encompassing 43,000 Km² which represents about 40% of this state. In addition to potable water supply for public consumption, this hydrographic basin is used for agricultural activities, such as fruit growing, shrimp farming, and fish culture. This basin supplies potable water to 46 cities in the state of Rio Grande do Norte (RN) and 102 cities in the state of Paraíba (PB), Brazil. The water supply mainly originates from two important reservoirs, the Engenheiro Armando Ribeiro Gonçalves (EARG) reservoir in RN, with a maximum capacity of 2.4 billion cubic meters and the Curema-Mãe D'água reservoir in PB, with 1.3 billion cubic meters capacity, considered strategic to the socioeconomic and environmental development of these states. 

The River Assu (05° 39′ 92′′ S and 036° 53′ 92′′ W) is one of the main rivers belonging to the Piranhas-Assu hydrographic basin. The relatively low depth (15 m) and high turbulence, caused by resuspension of organic sediments from the bottom layers, result in large amounts of dissolved humic compounds. These influence the water transparency and reduce the euphotic zone of this environment.

### 2.2. Sample Collection

Fishing trials were conducted with the help of local fishermen in the shallow stretches of the River Assu. Fish samples were captured on a monthly basis from May 2008 to June 2009, using gill and cast nets, with mesh size of 7 cm, with an effort of 2 hours per fishing trial.

Fish captured were numbered, measured, weighed, and samples of fish were used for morphometric measurements and meristic counts to confirm the taxonomical identification of the study species. Measures of total length (Lt ±1 cm), (the distance from the anterior extremity of the maxilla to the final extremity of the caudal fin), standard length (Ls ± 1 cm), (the distance between the anterior extremity of the maxilla and the last lumbar vertebra, total body mass (Wt ± 1 g), and gonad weight (Wg ± 0.1 mg) were recorded. Sample specimens of this species were deposited in the museum collection of the Department of Systematics and Ecology of the Federal University of Paraíba, Brazil.

The total length and weight were determined using the absolute frequencies of males and females (mean ± SD) in five classes of total length (Lt) and six of total weight (Wt). After a longitudinal incision in the abdomen of each fish sample, the gonads were removed. The macroscopic aspects and maturation stages of the gonads were observed besides determining the sex of each fish. 

The body length at first gonadal maturity (*L*_50_) where 50% of the individuals exhibited maturing gonads was estimated from the relative frequency distribution of adult males and females, using their standard length classes (Ls mean ± SD). 

Two methods were used to assess the reproductive period of the study species. The qualitative method was based on the monthly changes of macroscopic gonadal development stages and the quantitative method on monthly variations in parameters related to sexual maturation, such as gonadosomatic index (GSI) and condition factor (*K*). The gonadosomatic index was calculated as follows. GSI = (Wg/Wt) × 100, where Wg is gonad weight, and Wt is total body weight [11]. The condition factor (*K*) was determined as follows, where *K* = 100(Wt/Lt^*b*^); Wt = total body weight; Lt = total length; *b* = angular coefficient [12]. 

To determine the mean absolute fecundity, ten mature females were used, with mean length of 27.43 ± 2.98 cm and mean weight of 362.25 ± 75.08 g. The ovaries were removed, weighed, and preserved in modified Gilson solution for 24 hours for complete dissociation of oocytes, which were then washed and preserved in 70% ethyl alcohol. The mature ovaries weighed on an average 48.09 ± 26.76 g. A 10% sample was removed for counting the mature oocytes, and the values were extrapolated to 100%. 

Monthly and annual rainfall data (mean ± SD) were obtained from EMPARN (Agriculture Research Company of Rio Grande do Norte). The data were correlated with the GSI and K of *P. brevis *to determine the reproductive period.

Limnological variables, such as water temperature (°C), concentration of dissolved oxygen (mgL^−1^), pH, and electrical conductivity (*μ*Scm^−1^) (mean ± SD) were measured in situ, between 9:00 h and 10:00 h, using specific probes and a WTW multi-340i meter.

The *t*-test was used to determine the statistically significant differences between the lengths and weights of both sexes. Sexual proportion was calculated on a monthly basis, and the 1 : 1 null hypothesis was tested using the chi-square (*χ* ^2^) test. Principal component analysis (PCA) was applied to reduce the data size and order and identify the main environmental variables (rainfall, water temperature, concentration of dissolved oxygen, conductivity, and pH) related to the reproductive period of the study species. The ordinations were carried out using the software XLSTAT 7.5.3, at a significance level of *α* = 0.05. 

## 3. Results

**Figure 1:**
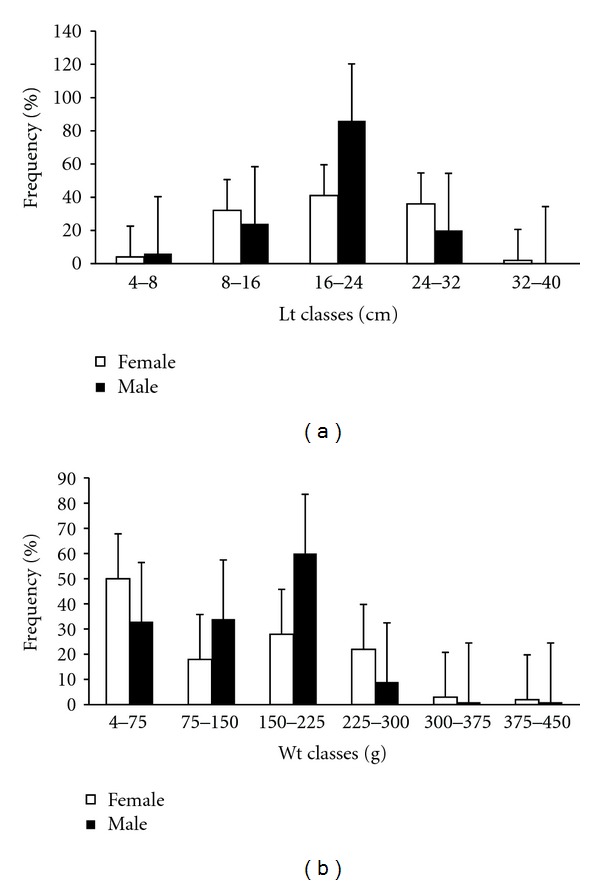
Monthly distribution of frequencies of males and females by total length (a) total weight and (b) with standard deviations.

A total of 257 specimens of *P. brevis* were captured. Analysis of total length and weight for both sexes showed that the smallest specimen captured during the study period was 5.3 cm in length and 5 g in weight, while the largest was 32.8 cm long and weighed 416.5 g. There were 121 females (19.2 ± 6.5 cm Lt and 134.3 ± 101.5 g Wt) and 136 males (20 ± 5.3 cm Lt and 139.3 ± 76.6 g Wt). Female body lengths ranged between 6.5 and 32.8 cm and body weights between 5 and 416.5 g, with predominance in the 16–24 cm and 4–75 g ranges. Male body lengths varied from 5.3 to 28.7 cm and body weights from 5 to 385.0 g, with predominance in the 16–24 cm and 150–225 g ranges (Figure 1). The differences between the lengths and weights of males and females were not statistically significant (*t* = −1.03243; *P* > 0.05; *t* = 0.450915; *P* > 0.05).

**Figure 2:**
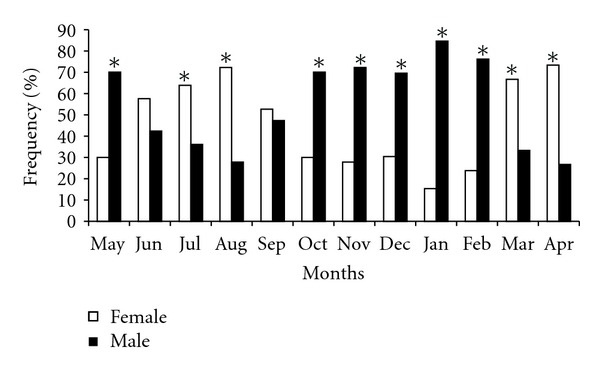
Monthly sex ratio of *Prochilodus brevis* (*χ ^2^, *P* < 0.05).

Monthly frequency distribution of males and females of *P. brevis *during the study period showed that the sex ratio was 1.3M : 1F thus differing from the expected 1M : 1F. This difference was statistically significant (*χ* ^2^ = 101.49; *P* < 0.00), except in June and September (*χ* ^2^ = 1.15  and 0.14). There was predominance of males, mainly from October to February and in May, while females predominated in March–April and July–August (Figure 2).

### 3.1. Gonad Maturation Stages

**Figure 3:**
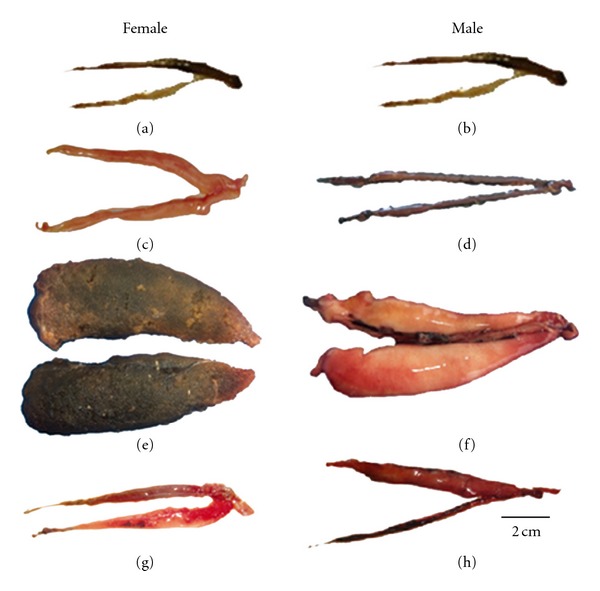
Female and male gonads of *Prochilodus brevis, *where (a) and (b) represent immature; (c) and (d) maturing; (e) and (f) mature; (g) and (h) spent gonads.

*P. brevis *did not exhibit any secondary sexual characteristics during the reproductive period, and hence no sexual dimorphism was observed. As such, it was possible to identify the sex of each individual only after the gonads were exposed. The following macroscopic stages of gonadal maturation were identified for both sexes: immature, maturing, mature, spent, and resting (Figure 3).

Immature females (*N* = 42) showed transparent filament-shaped ovaries (mean weight of 1.51 g), without any evidence of vascularization, with translucent cells in the central part and with smooth margins. In males (*N* = 41), the testicles (mean weight of 0.86 g) were translucent, small and thin, with undulating margins. 

In maturing females (*N* = 25), the ovaries were voluminous (weighing an average of 2.49 g), dark red in color with clearly visible vascularization. The testicles (*N* = 34) were thread-shaped, varying from translucent to slightly whitish (with mean weight of 0.84 g). 

Mature ovaries (*N* = 3) exhibited maximum size (with mean weight of 16.49 g) and occupied nearly all the coelomic cavity. They were reddish-brown in colour with vitellogenic oocytes visible to the naked eye. The testicles (*N* = 13) were clearly more developed than in the previous phase (with mean weight of 2.09 g). They were light pink in color and spermatic fluid flew under light pressure.

In the spent stage (*N* = 13), the ovaries were partially empty (mean weight of 0.79 g), reddish in colour with hemorrhaging aspects. The spent testicles (*N* = 17), (weighed an average of 1.02 g), were empty and translucent, showing hemorrhagic or flaccid aspects and were dark brown to reddish in colour in some of the regions.

The resting stage showed similar characteristics for both sexes, where the gonads occupy a small portion of the coelomic cavity. They showed foliaceous aspect, little vascularization with mean weight of 0.89 g for females (*N* = 40) and 0.52 g for males (*N* = 31). Specimens at resting stage could be distinguished from immature individuals by their relatively larger body size. 

**Figure 4:**
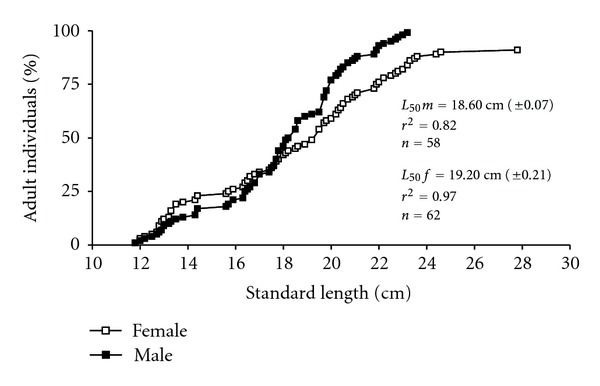
Size at first sexual maturity of females and males of *Prochilodus brevis*.

Body length at first gonadal maturity (*L*_50_) was 19.2 ± 0.21 cm for females (*N* = 62) and 18.6 ± 0.07 cm for males (*N* = 58) (Figure 4).

**Figure 5:**
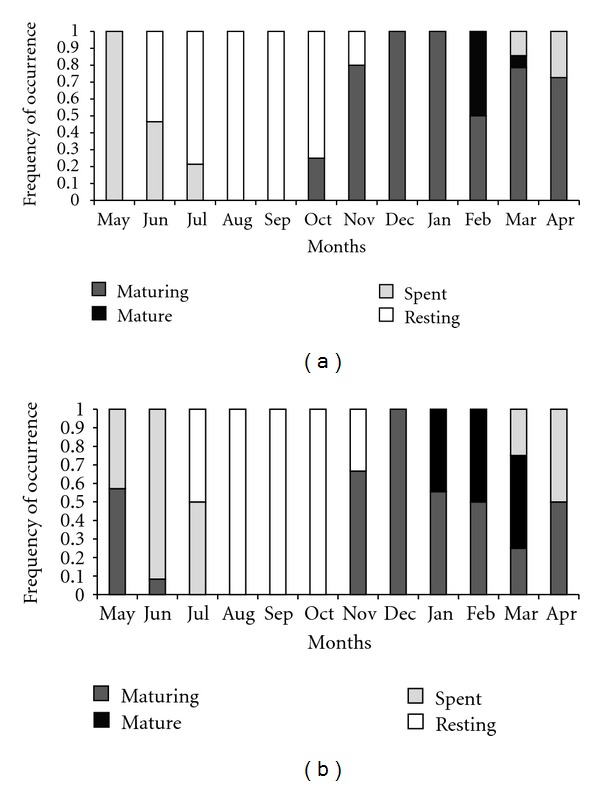
Monthly frequency of occurrence of gonad maturation stages in females (a) and males (b) of *Prochilodus brevis*.

Monthly frequency of gonadal maturation stages in *P. brevis* showed that maturing females appeared from October to April and males from November to June. Mature females were captured in February and March and males between January and March. Spent individuals of both sexes were found from March to July, while specimens in the resting phase occurred from June to November for females and July to November for males (Figure 5).

**Figure 6:**
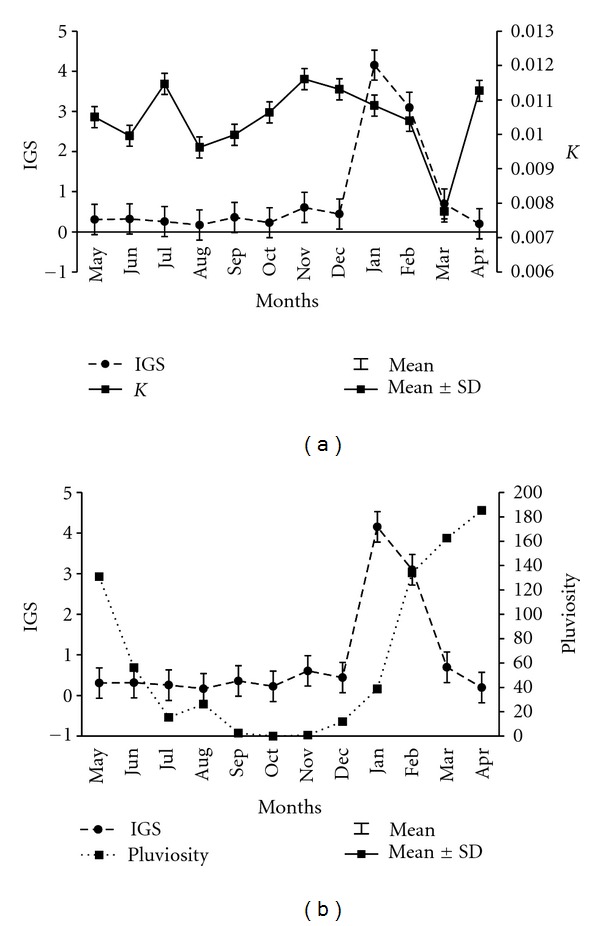
Monthly gonadosomatic index and condition factor of *P. brevis* (a) and gonadosomatic index of *P. brevis* and rainfall (b).

In both sexes, the mean GSI values started to increase in December, reaching a maximum in January (GSI = 4.15 ± 4.08), this was followed by a decrease, reaching a minimum in March (GSI = 0.70 ± 0.59). Specimens of both sexes in the spent stage were captured between March and July (GSI = 0.36 ± 0.26). The specimens were in resting stage in November–December (GSI = 0.36 ± 0.25) (Figure 6(a)).

Analysis of condition factor (*K*) showed a similar annual pattern for males and females of *P*. *brevis*. Temporal variations in *K* demonstrated a low range, where the highest values occurred from October to December (*K* = 0.012 ± 0.001) and the lowest from January to March (*K* = 0.010 ± 0.003). There was an annual peak in November 2008 (*K* = 0.012 ± 0.007) and a minimum value in March (*K* = 0.008 ± 0.005). *K* and GSI were inversely correlated, since *K* exhibited its highest values when GSI was lowest and vice versa (Figure 6(a)). 

Mean monthly rainfall data for the period of May 2008 to June 2009 showed that the rainy season in the semiarid region extends from January to June, with an average rainfall of 118 ± 58.3 mm. Intense rainfall occurred in March and April, reaching a peak of 185 mm in April. The dry season occurred from July to December, with mean rainfall of 9.5 ± 10.4 mm. There was no rain during the months of October and November. 

The gonadosomatic index for both sexes in relation to monthly mean rainfall were positively correlated (*r* = 0.11 and *P* < 0.05). The fish were ready to reproduce at the onset of the rainy season when their GSI values were highest. During the months of intense rainfall GSI values were low indicating that spawning had occurred (Figure 6(b)).

Absolute fecundity showed an amplitude of variation in the number of vitellogenic oocytes, ranging between 27, 454.93 and 140, 522.86, with a mean of 75,942.41 ± 62.12 mature oocytes.

The concentration of dissolved oxygen in May and June ranged from 4.8 to 10.1 mgL^−1^ (with an average of 7.49 ± 1.78), electrical conductivity from 175 to 1997 *μ*S cm^−1^ (mean of 448.93 ± 506.10), pH from 5.8 to 9.8 (mean of 7.98 ± 1.06), and temperature from 27 to 31.4°C (mean of 28.72 ± 2.07).

**Figure 7:**
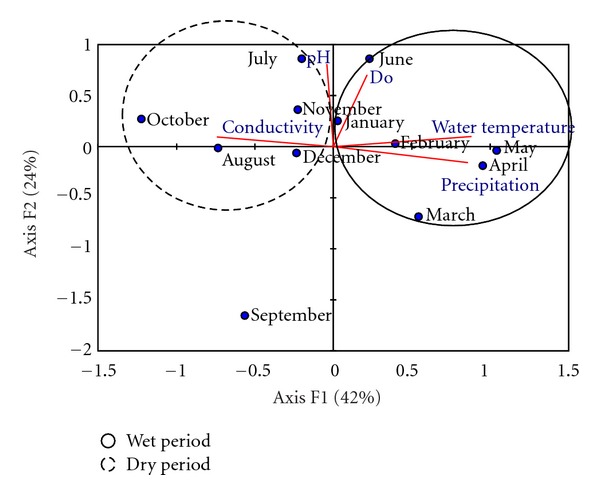
Principal component analysis of limnological variables during the rainy and dry seasons.

The first two axes of principal component analysis (PCA) explained 66% of limnological data variability. Water temperature and concentration of dissolved oxygen were the most marked factors during the rainy season, whereas electrical conductivity and pH were prominent during the dry season (Figure 7).

## 4. Discussion

Reservoirs were constructed on main hydrographic basins in order to supply drinking water, electrical energy, for agriculture and fish culture purposes. Despite their importance for human societal needs, the reservoirs caused serious irreversible alterations to the natural hydrological regime of rivers by altering ecosystem quality and the entire biota dynamics [13]. Fish population studies are important tools in establishing conservation and restoration programs in both anthropogenically altered and natural environments, since, besides being biologically significant, they can be easily incorporated into mathematical models of population assessments [14]. The present study could contribute information regarding the reproductive aspects of *P. brevis* that could be used in the management and conservation of this species. 

Females were predominant in the larger class sizes, showing higher body growth than the males. Larger females were also recorded for another species *Prochilodus affinis* in the São Francisco River basin of northeastern Brazil [15]. Probably the large body size and large ovaries of females are related to the reproductive success of this species.

The sex ratio differed from the expected 1 : 1 with a slight but significant predominance of males. Spatial sexual segregation may occur in the environment with males and females inhabiting different areas, which could explain the sex ratio observed in this study. Observation during the reproductive period indicated that the mature females were found near the river margins, whereas the males were in the deeper parts of the river. 

The length at first gonadal maturity (*L*_50_) estimated for *P. brevis* is different from those observed for other species of the same genus or family. For the related species *Curimatella lepidura* (Curimatidae) in the Juramento reservoir, Brazil, length at first gonadal maturity was 7.7 cm for females and 7.1 cm for males [16]. Generally the size at first maturity corresponds to the size when 50% of the population is composed of adult individuals. Size at first gonadal maturity is a very sensitive parameter in the life cycle of animals, and the considerable influence of the genetic component on the delimitation of this parameter suggests that it may be an important adaptive character [17]. This information becomes highly relevant in stock assessment models and also as a reference for regulating fishing. 

The reproductive characteristics and spawning period of the fish vary according to species and ecological characteristics of the drainage basins. The semiarid region of northeastern Brazil is characterized by a brief spell of rainy season coupled with a long dry season. Rain is the most important environmental factor that modulates the reproductive period of fish [9], and a similar trend is registered in this study. The GSI is a good indicator of the reproductive period of *P. brevis* and shows an inverse correlation with the condition factor. Males display a similar strategy to that of females in relation to gonad maturation and variations in the condition factor during the reproductive period [18]. 

Climatic changes have significant influence on ecological processes both directly and indirectly [19]. A number of complementary processes may act on fish populations in aquatic ecosystems, such as indiscriminate exploitation of fish stocks during breeding season and annual alterations in rainfall. These factors have been observed frequently in the semiarid region of northeastern Brazil.

Out of season fishing (during the reproductive period of fish), prohibited by IBAMA (Brazilian Institute of Environment) is steadily increasing year by year. Dry periods devoid of rain are becoming longer, and on the other hand, there are high levels of atypical rainfall over short periods. This is due to the influence of the climate phenomenon known as La Nina which occurred in 2008 and 2009. Fishing for *P*. *brevis* in the drainage basins is prohibited by IBAMA from May to July. However, this period should be reconsidered, given that the rainfall regime in the semiarid region of the state has been changing annually, causing modulations in the reproductive period of the species under study. We suggest that the prohibited fishing period be extended from March to July, thereby enabling the reproductive cycle of this species to be completed. However, this should be verified on an annual basis. 

Fish fecundity is a characteristic that can vary substantially in a species according to the length or weight [20]. In the present study, absolute fecundity values were higher due to the greater gonad and body size of *P. brevis*.

During the rainy season, the two important variables were the water temperature and the concentration of dissolved oxygen, which were inversely correlated. High electrical conductivity and alkaline pH values were recorded during the dry season. Similar results were observed for aquatic environments in the semiarid region [9]. 

*P. brevis* is an important ecological component of South American rivers. Annihilation of a fish species from this order may have a large impact on the ecological cycle. In the semiarid region, this species reproduces coinciding with beginning of rainfall; however, indiscriminate fishing occurs during this period. There is a need to reconsider the out of season fishing period, taking into consideration the intensification of climatic phenomena that is affecting the semiarid region of Brazil.
